# Phenolic Compounds Characterization and Biological Activities of *Citrus aurantium* Bloom

**DOI:** 10.3390/molecules17021203

**Published:** 2012-01-30

**Authors:** Ehsan Karimi, Ehsan Oskoueian, Rudi Hendra, Armin Oskoueian, Hawa Z. E. Jaafar

**Affiliations:** 1 Department of Science, Science and Research Branch, Islamic Azad University, Tehran 14515, Iran; 2 Department of Chemistry, Faculty of Mathematic and Natural Sciences, University of Riau, Pekanbaru, Riau 28143, Indonesia; 3 Department of Agronomy, Faculty of Agriculture, Islamic Azad University, Mashhad Branch, Mashhad 91735-413, Iran; 4 Department of Crop Science, Faculty of Agriculture, University Putra Malaysia, Serdang, Selangor 43400, Malaysia

**Keywords:** *Citrus aurantium* bloom, HPLC, antioxidant properties, anti-inflammatory and anticancer activity

## Abstract

*Citrus* plants are known to possess beneficial biological activities for human health. In addition, ethnopharmacological application of plants is a good tool to explore their bioactivities and active compounds. This research was carried out to evaluate the phenolic and flavonoid analysis, antioxidant properties, anti inflammatory and anti cancer activity of *Citrus aurantium* bloom. The total phenolics and flavonoids results revealed that methanolic extract contained high total phenolics and flavonoids compared to ethanolic and boiling water extracts. The obtained total phenolics value for methanolic *Citrus aurantium* bloom extract was 4.55 ± 0.05 mg gallic acid equivalent (GAE)/g dry weight (DW), and for total flavonoids it was 3.83 ± 0.05 mg rutin equivalent/g DW. In addition, the RP-HPLC analyses of phenolics and flavonoids indicated the presence of gallic acid, pyrogallol, syringic acid, caffeic acid, rutin, quercetin and naringin as bioactive compounds. The antioxidant activity of *Citrus aurantium* bloom were examined by the 1,1-diphenyl-2-picryl-hydrazyl (DPPH) assay and the ferric reducing/antioxidant potential (FRAP). The free radical scavenging and ferric reducing power activities were higher for the methanolic extract of *Citrus aurantium* bloom at a concentration of 300 μg/mL, with values of 55.3% and 51.7%, respectively, as compared to the corresponding boiling water and ethanolic extracts, but the activities were lower than those of antioxidant standards such as BHT and α-tocopherol. Furthermore, the anti-inflammatory result of methanolic extract showed appreciable reduction in nitric oxide production of stimulated RAW 264.7 cells at the presence of plant extract. Apart from that, the anticancer activity of the methanolic extract was investigated *in vitro* against human cancer cell lines (MCF-7; MDA-MB-231), human colon adenocarcinoma (HT-29) and Chang cell as a normal human hepatocyte. The obtained result demonstrated the moderate to appreciable activities against all cell line tested and the compounds present in the extracts are non-toxic which make them suitable as potential therapeutics.

## 1. Introduction

Nowadays, the study of oxygen-containing free radicals in humans and their roles has been a growing interest among scientists. The conclusion has been that these radicals may contribute as factors in decreasing the immune system function [1]. Synthetic antioxidants such as butylated hydroxytoluene (BHT) and butylated hydroxyanisole (BHA) are known to posses free radical inhibition properties in the human body, but these compounds can also be toxic and present hazards to the human body as well [2]. Fruits and vegetables are rich of secondary metabolites such as phenolics which are now identified as natural antioxidant agents. Phenolic compounds have been shown to possess an antioxidant activity based on their (hydroxyl group) donation to free radicals. Moreover, phenolic compounds also possess a wide spectrum of biological activities such as antimutagenic, anticarcinogenic, anti-inflammation, antiallergic, as well as the ability to modify gene expression [3,4,5,6,7,8,9,10].

*Citrus* is one of larges species among plant; it consists of 40 species which are distributed in all continents [11]. *Citrus* is one of the most important fruits, which is consumed mostly fresh and has been used as a herbal medicine or additive or food supplement. *Citrus* is believed to possess bioactivities such as antioxidant, anti-inflammatory, antimicrobial, and is suggested to be responsible for the prevention of cancer and degenerative diseases [12]. Those bioactivities of citrus are due to the present of bioactive compound such as phenolics, flavonoids, essential oil, and vitamins [13]. Bloom of *Citrus aurantium* has long history of usage and is believed to alleviate the heart diseases, anti-depressant and tonic among people living in the north of Iran. Information about possible bioactivities of this part of the plant is rather limited, therefore an experiment was conducted to determine phytochemical analysis including phenolic and flavonoid compounds and their biological activities such as antioxidant properties, anti inflammatory and anticancer activities.

## 2. Results and Discussion

### 2.1. Total Phenolic and Flavonoid Contents

The results of total phenolic and flavonoid contents are shown in Table 1. The results obtained showed that methanolic extract of citrus bloom contains high total phenolic and flavonoid contents compared to the ethanolic and hot water extract, with a value of 4.8 ± 0.05 mg gallic acid equivalent/g DW and 4.1 ± 0.05 mg rutin equivalent/g DW. The total phenolic contents obtained was found to be lower compared to total phenolic content from *C. aurantium* peel, with a value of 223.2 mg gallic acid equivalent/g while the total flavonoid contents were found to be lower, with a value of 7.7 mg quercetin equivalent/g [14]. Previous studies have reported that solvents such as methanol, ethanol, acetone, ethyl acetate in combination of water have usually been used for the extraction of phenolic and flavonoid contents from plants [15]. Furthermore, the polarity of solvent are also one of interest in the processing of phenolics and flavonoid extraction [16]. Due to the mentioned reasons, it is difficult to choose a standard extraction method suitable for the extraction of phenolic and flavonoids from plants. Usually, the least polar solvents are considered to be suitable for the extraction of phenolic and flavonoid contents. The results in Table 1 indicated that methanol was a highly efficient solvent to extract phenolic and flavonoid contents among the solvents used in this study. The findings were in agreement with Perez *et al*. [17] who found that ethanol and water were less efficient solvents for extracting phenolic compounds compared to methanol. Moreover, Karimi *et al*. [18] and Oskoueian *et al*. [19] also observed the highest phenolic, flavonoid and antioxidant activity in the crude methanolic extract of saffron stigma and *Jatropha curcas* leaf, root and stem bark as compared to ethanol and water. Therefore, this research shows that different extracting solvents influenced different levels of total phenolic and flavonoid compound in present study. 

**Table 1:** Total phenolic and flavonoids content of *Citrus aurantium* bloom.

Solvent	Phenolic Content ^1^	Flavonoid Content ^2^
Ethanol	4.55 ± 0.005 ^b^	3.83 ± 0.05 ^b^
Water	3.93 ± 0.58 ^c^	1.88 ± 0.01 ^c^
Methanol	4.83 ± 0.05 ^a^	4.11 ± 0.05 ^a^

^1^ mg gallic acid equivalent/g DW; ^2^ mg rutin equivalent/g DW; n = 3. Means within the same column with different letters are significantly different (*p* < 0.05).

### 2.2. Determination of Phenolic and Flavonoid Compounds by HPLC

**Figure 1:**
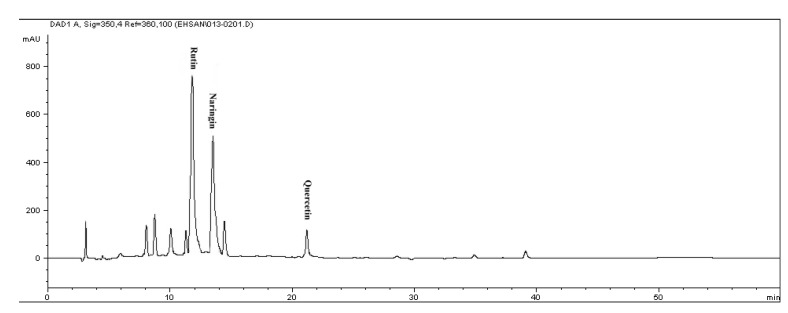
The RP-HPLC chromatogram of flavonoid compounds in methanolic extract of *Citrus aurantium* bloom. Identified compounds: Rutin, naringin and quercetin.

**Figure 2:**
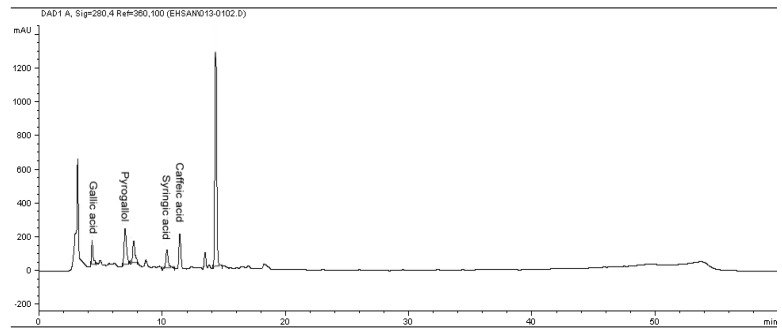
The RP-HPLC chromatogram of phenolic compounds in methanolic extract of *Citrus aurantium* bloom*.* Identified compounds: Gallic acid, pyrogallol, syringic acid and caffeic acid.

**Figure 3:**
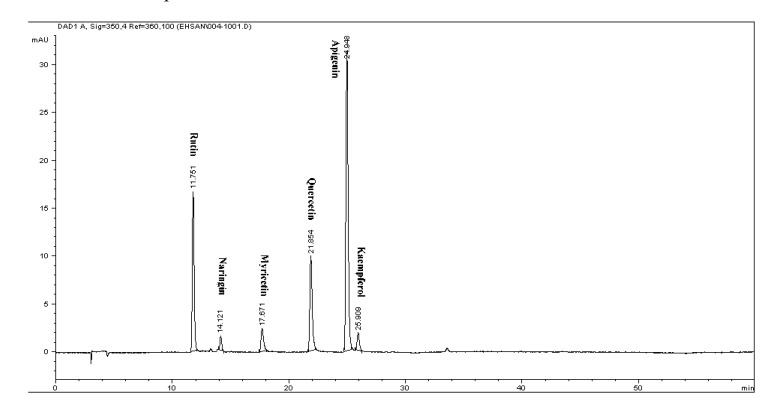
Chromatogram of flavonoids mixture of the standards detected at 350 nm by RP-HPLC: 11.76-rutin, 14.12-naringin, 17.67-myricetin, 21.854-quercetin, 24.85-apigenin and 25.909-kaempferol.

Reversed-phase (RP) chromatography was used to determine of phenolic and flavonoid compounds presented in citrus extract. The phenolic and flavonoid compounds were identified based on their retention times and quantified according to respective standard calibration curves (Figure 1 and Figure 2). The HPLC chromatogram revealed that gallic acid, pyrogallol, syringic acid and caffeic acid were the major phenolic compounds present in *C. aurantium* bloom with values of 212.4 ± 0.02 µg/g DW, 541.27 ± 0.03 µg/g DW, 269.04 ± 0.05 µg/g DW, and 249.9 ± 0.05 µg/g DW, respectively, while rutin, quercetin and naringin were detected as the major flavonoid compounds, with values of 362.8 ± 0.02, 185.37 ± 0.11 and 688.1 ± 0.05 µg/g DW (Table 2 and Table 3). Figure 3 shows a chromatogram of a mixture of standards. These findings are supported by Peleg *et al.* [20] who reported the presence of gallic acid, caffeic acid in several species of citrus. This result revealed that gallic acid, and other phenolic and flavonoid compounds may be responsible for the antioxidant activity in this plant and suggested that there seemed to be a good correlation among these compounds and antioxidant activity and other biological activity in this plant.

**Table 2:** Phenolic compounds of methanolic extract of *Citrus aurantium* bloom.

Sample	Phenolic contents (µg/g dry sample)					
	Gallic acid	Pyrogallol	Salicylic acid	Caffeic acid	Vanillic acid	Syringic acid
*C. Aurantium* bloom	212.42 ± 0.02	541.27 ± 0.03	ND	249.95 ± 0.05	ND	269.04 ± 0.05

ND: not detected.

**Table 3:** Flavonoid compounds of methanolic extract of *Citrus aurantium* bloom.

Sample	Flavonoid contents (µg/g dry sample)					
	Apigenin	Kaempferol	Myricetin	Naringin	Quercetin	Rutin
*C. Aurantium* bloom	ND	ND	ND	688.11 ± 0.05	185.37 ± 0.11	362.85 ± 0.01

ND: not detected.

### 2.3. Antioxidant Activity Determination

**Figure 4:**
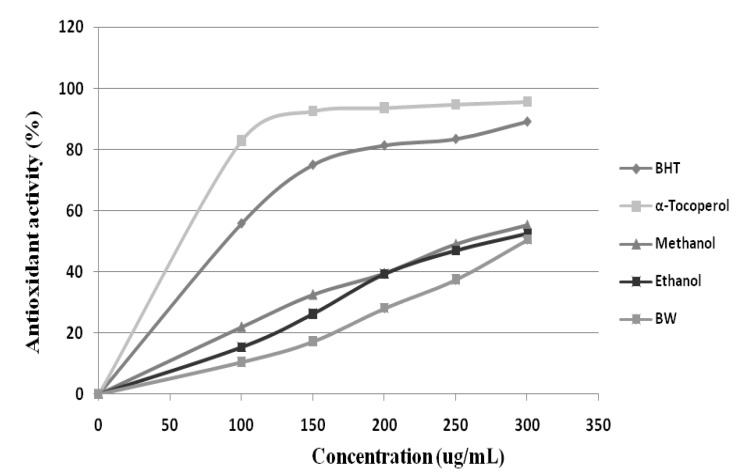
Free radical scavenging activity of *Citrus aurantium* extracts using different solvents by 1,1-diphenyl-2-picrylhydrazyl radicals. n = 3.

**Figure 5:**
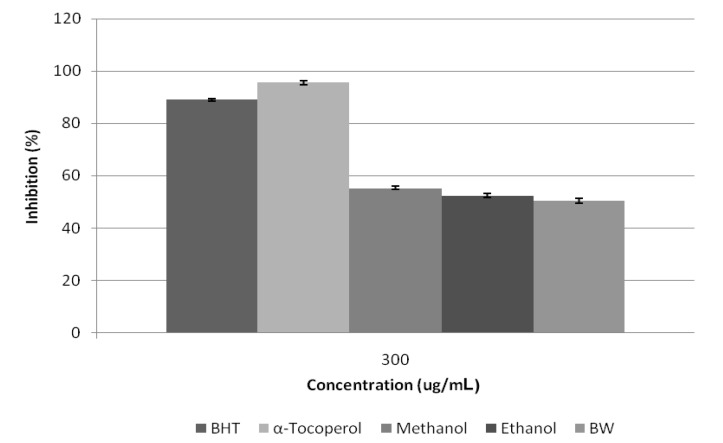
The free radical scavenging of *Citrus aurantium* bloom extracts using different solvents and reference antioxidants at 300 μg/mL.

Antioxidant research is a key topic in both the medical and food industry today. Antioxidants protect the body from reactive species. Previous researches have indicated the linear positive correlation between bioactive compounds of plant materials with their antioxidant capacities [21]. The antioxidant activities of *Citrus aurantium* Bloom determined by free radical scavenging activity (DPPH) and FRAP assay methods using different solvent polarity. The reduction of DPPH radical was followed by monitoring the decrease of absorption of sample extracts at 517 nm. The results obtained (Figure 4 and Figure 5) from DPPH radical-scavenging activity of *C. aurantium* bloom showed that all extracts exhibited DPPH radical inhibition activity at different concentrations. It was observed that methanolic extract show higher DPPH free radical scavenging activity compared to ethanol and hot water, with values of 55.32%, 52.41%, and 50.46%, respectively, but less than those of BHT and α-tocopherol at 300 µg/mL. Like the DPPH results, *C. aurantium* appeared to be active in the reduction of Fe^3+^. The ferric reducing power activity of *C. aurantium* bloom varied among the extracts, but the values were all lower than those of the standards. Methanolic extract showed a higher reductive potential than the boiling water and ethanolic extracts. The reductive potential of *Citrus aurantium* bloom extracts and standards at a concentration of 300 µg/mL (Table 4) were found to be in the ascending order: Vitamin C > α-tocopherol > BHT > methanol > ethanol > boiling water, with respective values of 96.1%, 92.9%, 89.5%, 51.7%, 47.6% and 43.5%. 

**Table 4:** FRAP activity of *Citrus aurantium* extracts using different solvents (methanol, boiling waterand ethanol). BHT, α-tocopherol and vitamin C were used as positive controls.

	Solvent used for Extraction	FRAP (300 μg/mL)
	Methanol	51.7 ± 37.3 ^d^
*Citrus aurantium* Bloom	Water	43.5 ± 23.4 ^f^
	Ethanol	47.6 ±18.7 ^e^
	BHT	89.5± 11.2 ^c^
Control	α-tocopherol	92.9 ± 25.4 ^b^
	Vitamin C	96.1 ± 41.2 ^a^

All analyses were the mean of triplicate measurements ± standard deviation. Results expressed in percent of antioxidant power at 300 μg/mL. Means not sharing a common letter within a column were significantly different at *p* ≤ 0.05.

Phytochemicals are chemicals derived from plant sources. Plants contain hundreds of phytochemicals such as flavonoids and phenolic acids. Research indicates phytochemicals such as polyphenols have high antioxidant activity [22]. Free radicals are highly reactive and are generated in the body through normal cellular function and are believed to cause lipid oxidation leading to cellular membrane damage [23]. The antioxidant activity of *C. aurantium* bloom extract might be due to the present phenolic and flavonoid which have been explained above. Wang *et al*. [24] mentioned that phenolic such as phenolic acid and flavonoid have important role due to the ability to scavenge free radical. 

Flavonoids act as “radical-scavengers” so they categorized in powerful antioxidants groups against free radicals. This activity is attributed to their hydrogen-donating ability. Indeed, the phenolic groups of flavonoids serve as a source of a readily available “H” atoms such that the subsequent radicals produced can be delocalized over the flavonoid structure [25,26]. Rapisarda *et al.* [27] reported the antioxidant capacity of some varieties of pigmented oranges, including Moro, Sanguinella, Tarocco and Washington. All examined orange juices show an antioxidant capacity, due to total phenol amounts and to their ability to interact with the biomembrane; their antioxidant capacity seems to be widely influenced by the anthocyanin concentrations in the pigmented oranges juices. This study shows that the daily phenol intake, as orange juice, may represent an additional protection *in vivo* against cellular biomolecule oxidation.

### 2.4. Anti Inflammatory Activity

**Figure 6:**
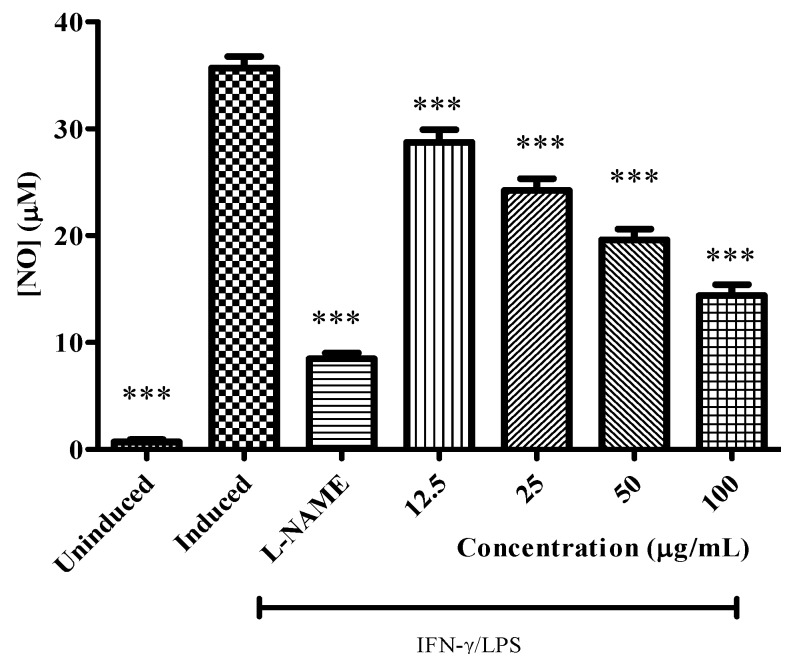
Effect of *C. aurantium* extracts at various concentration on the production of NO by LPS/IFN-γ stimulated RAW 264.7 cells. Each bar represents the mean ± standard error of mean from three independent experiments. *** *p* < 0.0001 indicates a significant difference from the LPS/IFN-γ stimulated cells analyzed by using the Dunnett’s Comparison Test.

**Figure 7:**
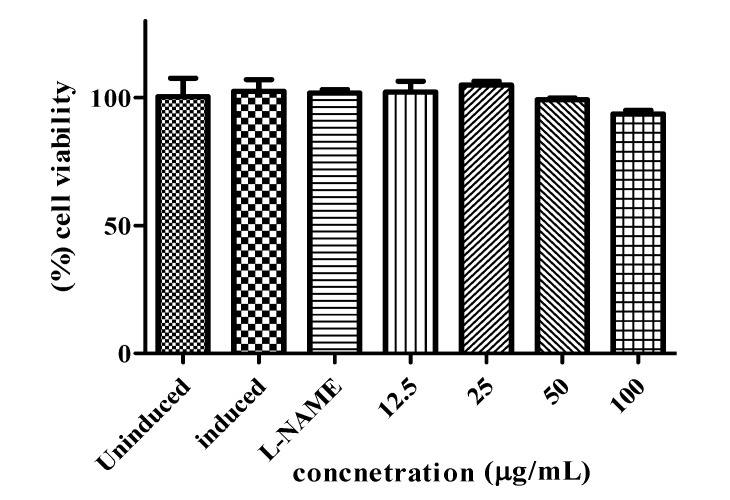
Effect of the *C. aurantium* extracts on cell viability of RAW 264.7 cells. Each bar represents the mean ± standard error of mean from three independent experiments.

The crude methanolic extracts of *C. aurantium* blooms were analyzed for their inhibitory effects on NO production from macrophages RAW 264.7 cells, induced by LPS and IFN-γ. Anti inflammatory results indicated that the production of nitric oxide was significantly inhibited by methanolic extract of *C. aurantium* of different concentrations (Figure 6). In the highest concentration (100 μg/mL), the value of nitric oxide production was 14.40 μM while this value was still higher comparing to positive control (L-NAME). Even though the nitric oxide inhibitory activity of the sample was lower than the positive control the inhibition value was but still an appreciable amount. Uninduced RAW 264.7 cells showed the lowest nitric oxide production, even though the cells had not been stimulated by LPS and IFN-γ low production of nitric oxide in normal condition. NO produced by constitutive NOS is vital for other physiological function such as neurotransmitter, blood flow, and synaptic plasticity. Furthermore, the cells without inhibitor showed the highest production of nitric oxide, this is due to the presence of inducible nitric oxide synthase (iNOS) inducers, which are LPS and IFN-γ in the absence of any iNOS inhibitor such as L-NAME or plant extract. The MTT assay was done accordingly to test the cell viability of RAW 264.7 cells while the nitric oxide production inhibited at different concentration by plant extract. As shown in Figure 7 the cell viability was not affected while different concentrations of plant extract were applied. At the highest concentration used (100 µg/mL) percentage of cell viability for the extracts was still more than 90% indicating the safety of respective plant extract. *C. aurantium* blooms contained the highest amount of flavonoid and appreciable level of phenolics (Table 1). Sumanont *et al.* [28] demonstrated that the mechanism of phenolic compounds in antioxidant activity and their ability to act as free radical scavengers resulting in to formation of phenoxyl radicals. Kazlowska *et al.* [29] and Oskoueian *et al.* [19] suggested that the inhibition of iNOS in the RAW 264.7 cell, is due to the suppressing action of flavonoids. Methanolic extract of *Parinari polyandra* (main compounds were flavonoids and tannins) and *Alchornea cordifolia* (main compounds were flavonoids and phenolics) revealed anti-inflammatory effects at concentration of 200 and 100 mg/kg paw oedema mice respectively [30,31]. Quercetin, hesperitin and morin at each concentration of 75 mg/kg exhibited anti-inflammatory effect in carrageenan-induced paw oedema mice [32].

### 2.5. Anti Cancer Activity

**Figure 8:**
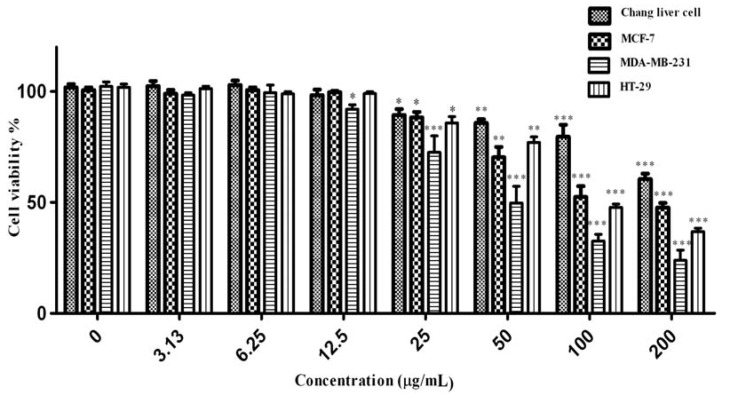
Effect of crude methanolic extract of *Citrus* blooms on Chang liver, MCF-7, MDA-MB 231 and HT-29 cells survival. All values represent the mean ± S.E.M from three independent experiments. *** *p* < 0.001, ** *p* < 0.01 and * *p* < 0.1 indicate significant difference compared to the untreated control group.

Cancer is a group of diseases characterized by cells that grow out of control; in many cases, they form masses of cells, or tumors, that infiltrate, crowd out, and destroy normal tissue. Consumption of antioxidant rich fruits and vegetables in our daily diets significantly reduces the risk of many cancer diseases, suggesting that confident antioxidants could be effective agents for the inhibition of cancer spread [33]. The obtained results of anti cancer activity of *C. aurantium* extracts are shown in Figure 8. Increase in extracts concentration of up to 200 µg/mL, could reduce the cell viabilities significantly (*p* < 0.001) in a dose-dependent manner in all four cell lines tested. The IC_50_ values of extracts used in this study are presented in Table 5. According to the US NCI plant screening program, a crude extract is generally considered to have *in vitro* cytotoxic activity if the IC50 value (concentration that causes reduction in cell viability to 50%) is less than 30 µg/mL [34]. 

**Table 5:** The IC50 values of extracts and positive control on Chang liver, MCF-7, MDA-MB 231 and HT-29 cell lines.

Sample	IC50 value (μg/mL)			
	Chang liver	MCF-7	MDA-MB 231	HT-29
*Citrus aurantium* Blooms	>200	152.34 ± 0.75	49.74 ± 0.75	96.23 ± 0.75
Tamoxifen	45.07 ± 2.59	17.31 ± 0.93	17.51 ± 0.25	18.11 ± 0.89

**Figure 9:**
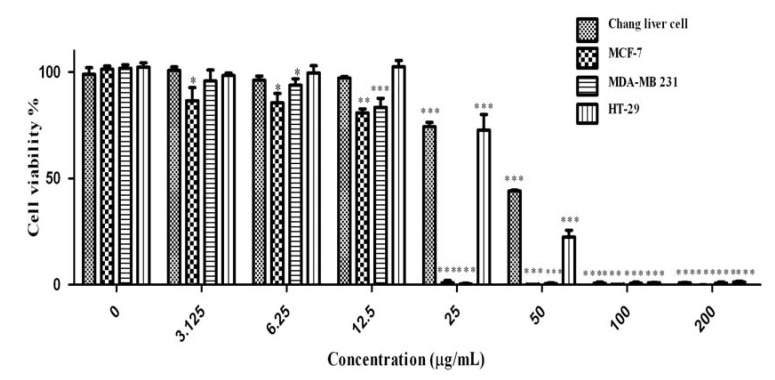
Effect of tamoxifen as a positive control on Chang liver, MCF-7, MDA-MB 231 and HT-29 cells survival. All values represent the mean ± S.E.M from three independent experiments. *** *p* < 0.001, ** *p* < 0.01 and * *p* < 0.1 indicate significant difference compared to the untreated control group.

Cytotoxicity of the methanolic extract is appeared to be more active on MDA-MB-231 compare with other cell line. Concentration of 50 µg/mL of methanolic extract decreased the MDA-MB-231 cell viability to 49.7% while the Chang liver cell viability was 85.8%. Tamoxifen was used as a positive control (Figure 9) in this study. The IC_50_ concentration of tamoxifen (Table 5) for HT-29, Chang liver cell, MDA-MB-231 and MCF7 were 18.11, 45.07, 17.51 and 17.31 µg/mL respectively. The activity of tamoxifen was found to be higher than that of *C. aurantium* extracts. All the extracts showed cytotoxic effects at various concentrations. Different bioactive compounds and variation of phytochemicals such as phenolics, flavonoids could lead to the cytotoxic activity of this medicinal plant. Mavundza *et al.* and Oskoueian *et al.* [19,35] indicated the ability of flavonoids and phenolic compounds to serve as anticancer agents. Zhang *et al.* [36] showed that kaempferol, quercetin, anthocyanins, coumaric acid and ellagic acid isolated from strawberry inhibited the growth of breast (MCF-7), oral (KB, CAL-27), colon (HT-29, HCT-116), and prostate (LNCaP, DU-145) human cancer cell lines. Similar results have also been reported in previous studies stating that polyphenols such as resveratrol, quercetin, catechin, and epicatechin isolated from wine extract [37] and green tea polyphenols like epigallocatechin and epicatechin [38] play important roles as anticancer agents [39,40,41,42,43].

## 3. Experimental

### 3.1. Plant Material

The petals of *Citrus aurantium* bloom were collected from the fully opened flowers of twenty trees located at three different gardens located in Ramsar, Mazandaran, Iran on April 2007. The sampleswere freeze-dried, ground to powder using a pestle and mortar. The ground powder was kept in 4 °C for further experiments.

### 3.2. Preparation of Extracts

The bloom of *Citrus* aurantium was extracted using three different solvents: ethanol, methanol and hot water. For methanolic and ethanolic extraction method of Crozier *et al.* [44] was followed with slight modification. Air-dried sample (0.5 g) weighed and placed into a 100 mL conical flask, and treated with 80% (v/v) ethanol or methanol (40 mL). It was followed by an addition of 6 M HCl (10 mL). The mixture was refluxed for 2 h at 90 °C and filtered by using Whatman No. 1 filter paper (Whatman, Welwyn Garden City, UK) continued by evaporation of filtrate using a vacuum rotary evaporator (Buchi, Flawil, Switzerland). The boiling water extraction was carried out according to a method of Gülçin *et al.* [45]. Five grams of ground citrus blooms were placed in a beaker glass and mixed with boiling water (100 mL) followed by magnetic stirring for 15 min. The extract was then filtered and evaporated as mentioned above. The dried crude extract was weighed and dissolved in methanol and stored at −20 °C for further experiments. 

### 3.3. Total Phenolic Content

The amount of total phenolic compounds in the citrus extract was determined with the Folin-Ciocalteu's reagent according to Ismail *et al.* [46] Results of total phenolic contents were expressed as milligrams of gallic acid equivalents (GAE) per gram dry weight (DW).

### 3.4. Total Flavonoid Content

Total flavonoid compound was measured by the aluminum chloride colorimetric assay based on Marinova *et al.* [3]. Total flavonoid compound of extracts were expressed as mg rutin equivalent/g dry weight (DW).

### 3.5. Determination of Phenolic and Flavonoid Compounds by HPLC

The phenolic and flavonoid compounds of citrus bloom quantitatively measured by reversed-phase high-performance liquid chromatography (HPLC) based on the method described by Crozier *et al.* [44] with some modifications. Phenolic compounds standards consisted of gallic acid, syringic acid, vanillic acid, salicylic acid, caffeic acid and the flavonoid compounds standards of quercetin, rutin, myricetin, kaempferol, naringin, apigenin, genistein, daidzein, and pyrogallol. An aliquot of sample extract was loaded on an Agilent-1200 series HPLC instrument equipped with a UV-Vis photodiode array (DAD) detector, binary pump, vacuum degasser, auto sampler and analytical column (Intersil ODS-3 5um 4.6 × 150 mm; Gl Science Inc, Tokyo, Japan). Solvents comprised deionized water and acetonitrile. The pH of water was adjusted to 2.5 with trifluoroacetic acid. The phenolic and iso-flavonoid compounds were detected at 280 nm and the flavonoid compounds at 350 nm. The column was equilibrated with 85% solvent A (water) and 15% solvent B (acetonitrile) then the ratio of solvent B was increased to 85% in 50 min followed by reducing solvent B to 15% in 55 min. this ratio was continued to 60 min for the next analysis with flow rate at 0.6 mL/min. All the standards were purchased from Sigma Chemical Company. 

### 3.6. Antioxidant Activity (DPPH Free Radical Scavenging Activity)

The free radical scavenging activity of the plant extracts was determined using the DPPH assay as described by Gülçin *et al*. [45]. One mL of each extract (methanolic, ethanolic and water) of *Citrus aurantium* petals at different concentration was mixed with 0.1 mM solution of 1,1-diphenyl-2-hydrazyl (DPPH) in methanol (3 mL) and incubated for 30 min in the dark condition, the absorbance of the mixture was read using a visible spectrophotometer (Novaspec II visblespectro) at 517 nm. BHT and α-tocopherol were used as antioxidant standards. Free radical scavenging activity from the sample was calculated according to the formula:

Free radical scavenging activity = [(A_0_ − A_1_)/A_0_] × 100%

where A_0_ was the absorbance of the control reaction and A_1_ was the absorbance in the presence of the sample.

### 3.7. Ferric Reducing Antioxidant Power (FRAP)

The ferric reducing property of the extracts was determined using an assay described by Yen and Chen [47]. The assay was carried out in triplicate. BHT, α-tocoferol and vitamin C were used as standard antioxidants.

### 3.8. Anti Inflammatory Assay

The murine monocytic macrophage cell line RAW 264.7 was cultured in Dulbecco’s Modified Eagle Media (DMEM; 2 mM L-glutamine, 45 g/L glucose, 1 mM sodium pyruvate, 50 U/mL penicillin; 50 µg/mL streptomycin) with 10% foetal bovine serum (FBS). The cells were cultured at 37 °C with 5% CO_2_ and were split twice a week. 1 × 10^6^ cells/mL RAW 264.7 cells were seeded in 96-well tissue culture plate and incubated for 24 h at 37 °C with 5% CO_2_. The cells were then incubated in prepared DMEM medium containing 100 µL of test extract in DMSO and serially diluted to give a final concentration of 100 µg/mL in 0.1% DMSO. Cells were then stimulated with 200 U/mL of IFN-γ and 10 µg/mL LPS for another 17 h. The presence of nitrite was determined in cell culture media by Griess reagent and absorbance was read at 550 nm using a microplate reader (Spectra Max Plus 384, Molecular Devices Inc., Sunnyvale, CA, USA). Nitrite concentration in the supernatants was determined by comparison with a sodium nitrite standard curve. The amount of cell viability was detected by MTT cytotoxicity assay. L-NAME was used as iNOS inhibitor (control) at concentration 250 µM [11]. 

### 3.9. Anti Cancer Activity Assay

Human cancer cell lines (MCF-7; MDA-MB-231), human hepatocytes (Chang liver cells) and human colon adenocarcinoma (HT-29) cell lines obtained from the American Type Culture Collection (ATCC) were used in this study. Cells were grown at 37 °C in humidified 5% CO_2_ and 95% air atmosphere in DMEM. Monolayers of the cells (5 × 10^3^/100 µL) were grown in 96-well microtitre plates and exposed to two-fold serial dilution of the extracts from 200 µg to 3.1 µg/100 µL. After 3 days incubation at 37 °C, the cytotoxicity of extracts was determined by using MTT assay according to Ahmad *et al.* [48,49]. Tamoxifen, which is a known anticancer drug, was used as a positive control in the present study.

### 3.10. Statistical Analysis

Data were subjected to one-way analysis of variance (ANOVA) using a complete randomized design following the model: Yi = μ + Ti + ei, where μ is the mean value, Ti is the treatment effect and ei is the experimental error, respectively. Differences in LSD were considered significant at *p* < 0.05. GraphPad Prism 5 software (GraphPad Software Inc., San Diego, CA, USA) was used for all the statistical analyses in anti-inflammatory assay [50,51,52,53].

## 4. Conclusions

*Citrus aurantium* bloom extracts contained phenolic and flavonoid compounds. The *Citrus aurantium* bloom extracts showed good antioxidant activity as measured by DPPH radical scavenging activity and ferric reduction power activity. The extracts also actively inhibited the iNOS in macrophages RAW 264.7 cell, induced by LPS and IFN-γ, indicating their potential as anti-inflammatory agent. The cytotoxicity assay indicated the potential of this medicinal plant as a source of anticancer therapeutic compounds.
